# Cold exposure alters lipid metabolism of skeletal muscle through HIF-1α-induced mitophagy

**DOI:** 10.1186/s12915-023-01514-4

**Published:** 2023-02-08

**Authors:** Wentao Chen, Ziye Xu, Wenjing You, Yanbing Zhou, Liyi Wang, Yuqin Huang, Tizhong Shan

**Affiliations:** 1 College of Animal Sciences, Zhejiang University, 866 Yuhangtang Road, Hangzhou, 310058 China; 2 Key Laboratory of Molecular Animal Nutrition (Zhejiang University), Ministry of Education, 866 Yuhangtang Road, Hangzhou, 310058 China; 3 Key Laboratory of Animal Feed and Nutrition of Zhejiang Province, 866 Yuhangtang Road, Hangzhou, China

**Keywords:** Cold exposure, Lipid remodeling, Mitophagy, Skeletal muscle, Lipidomic, Transcriptome

## Abstract

**Background:**

In addition to its contractile properties and role in movement, skeletal muscle plays an important function in regulating whole-body glucose and lipid metabolism. A central component of such regulation is mitochondria, whose quality and function are essential in maintaining proper metabolic homeostasis, with defects in processes such as autophagy and mitophagy involved in mitochondria quality control impairing skeletal muscle mass and function, and potentially leading to a number of associated diseases. Cold exposure has been reported to markedly induce metabolic remodeling and enhance insulin sensitivity in the whole body by regulating mitochondrial biogenesis. However, changes in lipid metabolism and lipidomic profiles in skeletal muscle in response to cold exposure are unclear. Here, we generated lipidomic or transcriptome profiles of mouse skeletal muscle following cold induction, to dissect the molecular mechanisms regulating lipid metabolism upon acute cold treatment.

**Results:**

Our results indicated that short-term cold exposure (3 days) can lead to a significant increase in intramuscular fat deposition. Lipidomic analyses revealed that a cold challenge altered the overall lipid composition by increasing the content of triglyceride (TG), lysophosphatidylcholine (LPC), and lysophosphatidylethanolamine (LPE), while decreasing sphingomyelin (SM), validating lipid remodeling during the cold environment. In addition, RNA-seq and qPCR analysis showed that cold exposure promoted the expression of genes related to lipolysis and fatty acid biosynthesis. These marked changes in metabolic effects were associated with mitophagy and muscle signaling pathways, which were accompanied by increased TG deposition and impaired fatty acid oxidation. Mechanistically, HIF-1α signaling was highly activated in response to the cold challenge, which may contribute to intramuscular fat deposition and enhanced mitophagy in a cold environment.

**Conclusions:**

Overall, our data revealed the adaptive changes of skeletal muscle associated with lipidomic and transcriptomic profiles upon cold exposure. We described the significant alterations in the composition of specific lipid species and expression of genes involved in glucose and fatty acid metabolism. Cold-mediated mitophagy may play a critical role in modulating lipid metabolism in skeletal muscle, which is precisely regulated by HIF-1α signaling.

**Supplementary Information:**

The online version contains supplementary material available at 10.1186/s12915-023-01514-4.

## Background

Skeletal muscle is a specialized thermogenic and metabolic organ in mammals. They play an essential role in maintaining thermal and metabolic homeostasis [1, 2]. Maintenance of skeletal muscle metabolism depends on mitochondria, which is the essential organelles for energy production in the body. The quality and function of mitochondria are particularly important for maintaining metabolic homeostasis in skeletal muscle. Enhanced mitochondrial quality results in improved whole-body metabolic homeostasis and increased energy expenditure, which has been recognized as an important strategy for combatting obesity and metabolic disorders in the last decade [3–5]. However, mitochondrial dysfunction and disruption of the mitochondrial structure due to environmental stress and chronic disease severely impair skeletal muscle mass and function [6–8]. Therefore, cells require sophisticated systems and fundamental mechanisms for maintaining mitochondrial fitness to meet environmental needs. Mitochondrial quality control relies on diverse pathways: reactive oxygen species (ROS) clearance, DNA repair, mitochondrial fusion and fission, and protein refolding/degradation [9]. Mitophagy plays an important role in eliminating damaged mitochondria to maintain cell structure and function. This process has been regarded as the major quality control mechanism for selective targeting and removal of damaged or dysfunctional mitochondria to ensure metabolic demands [10, 11]. Dysregulation of mitophagy has been implicated in the pathogenesis of many chronic diseases, including neurodegenerative diseases, metabolic disorders, and heart failure [12–14].

Acute cold exposure has been reported to increase energy expenditure and induce metabolic changes due to activated adaptive thermogenesis in the whole body [15–17]. Brown adipose tissue (BAT), as a major thermogenic organ, can contribute to improving lipid metabolism by regulating the uptake of glucose and fatty acids upon cold acclimation [18]. In addition, cold exposure also causes the formation of beige fat, also known as browning of white adipose tissue (WAT), which is considered to be an effective means of combating metabolic diseases and obesity [19]. A recent study shows that cold stress leads to dramatic changes in the overall lipid composition of WAT and transcriptional programs that are negatively correlated to metabolic diseases [20]. In addition to adipose tissue, skeletal muscle also has the capacity to induce energy-consuming futile cycles for both shivering and non-shivering thermogenesis [21]. Importantly, skeletal muscle plays a beneficial role in improving insulin sensitivity by regulating mitochondrial biogenesis and metabolic responses in patients with type 2 diabetes [22, 23]. However, how cold exposure regulates lipid metabolism in muscle and its molecular mechanisms are largely unclear.

Our previous results indicated cold exposure can regulate the lipid metabolism and fatty acid composition in the skeletal muscle of farmed animals by promoting fatty acid biosynthesis and decreasing mitochondrial beta-oxidation [24]. To better understand the regulation of lipid metabolism and mitochondrial activity in skeletal muscle during the cold environment, we examined total triglyceride (TG) levels and lipid class composition in skeletal muscle from mice following 3 days of cold exposure. We found cold exposure led to a significant increase in intramuscular fat deposition, which is associated with the composition of different lipid classes, especially in the levels of TG. RNA-seq and qPCR analysis showed that the expression of genes related to glucose and lipid metabolism altered significantly after cold exposure. In addition, autophagy and mitophagy in skeletal muscle were highly stimulated, which was accompanied by the downregulation of gene expression related to fatty acid oxidation and mitochondrial biogenesis. Mechanistically, we observed that HIF-1α signaling was activated in response to a cold challenge, which may contribute to intramuscular TG deposition and mitophagy in cold environments. Our findings demonstrate the critical role of mitophagy in thermogenesis and lipid metabolism in skeletal muscle during acute cold exposure.

## Results

### Cold stress promoted lipid droplet deposition in skeletal muscle

**Fig. 1:**
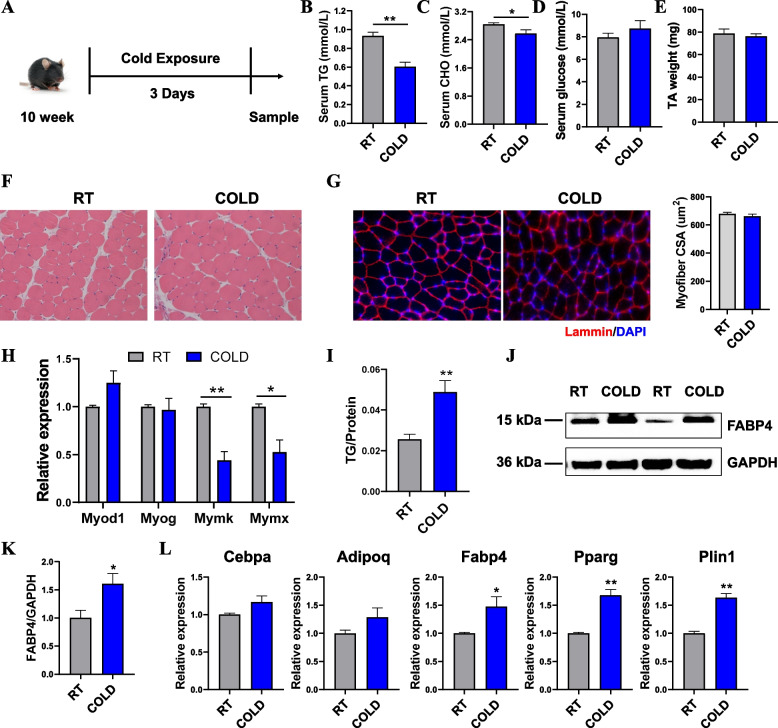
Acute cold exposure results in increased levels of triglycerides in skeletal muscle. **A** Experimental plan for short-term cold exposure. Muscle and adipose tissue were isolated from mice maintained at room temperature (RT, 23 °C) or cold (4 °C) for 3 days (*n* = 8). **B**–**D** Triglyceride (TG), cholesterol (CHO), and glucose levels in serum (*n* = 6). **E** TA muscle weight of the RT and COLD group mice at 10 weeks of age (*n* = 6). **F** Representative images of H&E staining of TA muscles of the RT and COLD group mice at 10 weeks of age. **G** Representative immunofluorescence staining of α-laminin in the TA muscle of the RT and COLD group mice at 10 weeks of age. **H** Quantitative PCR (qPCR) analysis of *Myod1*, *Myog*, *Mymk*, and *Mymx* in TA muscle during the cold challenge (*n* = 6). **I** TG content from TA muscle of the RT and COLD group mice at 10 weeks of age (*n* = 6). **J**–**K** Western blot of FABP4 protein levels and quantitative analysis in TA muscle during the cold challenge. **L** qPCR analysis of *Cebpa*, *Adipoq*, *Fabp4*, *Pparg*, and *Plin1* in TA muscle during the cold challenge (*n* = 6). Error bars represent s.e.m. *P < 0.05, **P < 0.01, ***P < 0.001, two-tailed Student’s *t*-test

In our previous study, acute cold exposure could induce changes in lipid composition patterns and transcriptome dynamics in adipose tissue, which may play a beneficial role in combating obesity [20]. Since skeletal muscle contributes to both adaptive and non-adaptive thermogenesis, we wanted to explore the adaptive changes and metabolic remodeling of skeletal muscle in response to cold exposure. In the present study, we maintained mice at either room temperature (RT, 22 °C) or in a cold environment (COLD, 4 °C) for 3 days (Fig. 1A). We found that cold exposure led to a significant decrease in serum TG and cholesterol (CHO) level, but had no significant effect on blood glucose changes (Fig. 1B–D). In addition, cold exposure resulted in body weight loss and reduced adipose tissue mass (Additional file 1: Fig. S1A, B). Hematoxylin-eosin (H&E) staining results showed that cold exposure induced inguinal WAT (iWAT) browning (Additional file 1: Fig. S1C). Consistently, cold exposure dramatically increased the uncoupling protein 1 (Ucp1) protein levels in WAT and BAT (Additional file 1: Fig. S1D, E). Additionally, mRNA levels for type II iodothyronine deiodinase (*Dio2*), peroxisome proliferator-activated receptor gamma coactivator 1α (Ppargc1α), cell death-inducing DFFA-like effector a (*Cidea*), PR domain containing 16 (*Prdm16*), and *Ucp1* were significantly elevated in iWAT upon 3-day cold exposure (Additional file 1: Fig. S1F, G). These results demonstrated that cold exposure led to increased fat mobilization in the body. Next, to determine whether cold exposure could result in changes in skeletal muscle development and metabolism, we examined different hind limb muscles. There was no significant change in the hind limb muscle mass of adult mice after short-term cold exposure, including tibialis anterior (TA), gastrocnemius (GAS), extensor digitorum longus (EDL), and soleus (SOL) muscles (Fig. 1E; Additional file 1: Fig. S1H). We then examined muscle fiber development and found no significant differences in the morphological analysis of TA muscle (Fig. 1F, G), which is often used to explore skeletal muscle development and regeneration [25]. In addition, the average cross-sectional area (CSA) of muscle fibers did not change significantly (Fig. 1G). Notably, cold exposure did not change the mRNA levels of the myogenic transcription factors *Myod1* and *Myog*, but decreased the expression of the fusion-related genes, *Mymk* and *Mymx* (Fig. 1H). Previous studies have shown that the increase in the extracellular muscle energy supply is mediated via an increase in the blood delivery of glucose, non-esterified fatty acids (NEFAs), and TG [26]. Hence, we hypothesized that reduced serum TG and CHO would be transported to muscle due to its adaptive thermogenesis during cold exposure. We first examined the TG content of the muscle and found a robust increase in intramuscular fat (IMF) deposition after cold exposure (Fig. 1I). In addition, cold markedly elevated the mRNA and protein expression levels of the fatty acid binding protein 4 (FABP4) (Fig. 1J, K). Moreover, the mRNA levels of fat deposition-related genes, including peroxisome proliferator-activated receptor gamma (*Pparg*) and perilipin 1 (*Plin1*), were significantly elevated (Fig. 1L). Together, these results suggest that short-term cold exposure resulted in increased muscle fat deposition.

### Cold stress induced changes in the overall composition and distribution of lipids in skeletal muscle

**Fig. 2:**
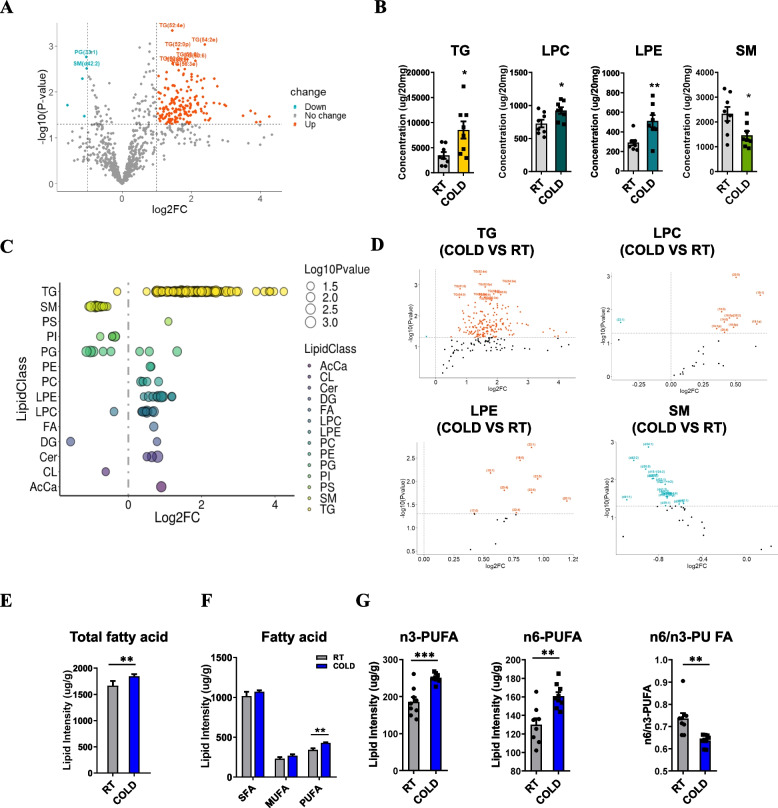
Changes in the overall lipid composition and distribution in TA muscle in response to cold exposure. **A** Volcano plot of lipid species altered in COLD vs. RT muscle (*n* = 8). The *x*-axis indicates the Log2 fold changes (log2FC) of all identified lipid species and the *y*-axis indicates the negative Log10 *P* value. **B** The intensity of TG, LPC, LPE, and SM in the TA muscle of the RT and COLD group mice (*n* = 8). **C** Log2 fold changes in significantly changed lipid species in the cold-treated versus RT mice and the corresponding significance values displayed as −log10 (*P* value) (*n* = 8). **D** Volcano plots of TG, LPC, LPE, and SM species altered in COLD vs. RT muscle (*n* = 8). **E** The intensity of total fatty acid in the TA muscle of the RT and COLD group mice (*n* = 8). **F** The intensity of SFA, MUFA, and PUFA in the TA muscle of the RT and COLD group mice (*n* = 8). **G** The intensity of n3-PUFA, n6-PUFA, and the percentages of n6-PUFA/n3-PUFA in the TA muscle of the RT and COLD group mice (*n* = 8). Error bars represent s.e.m. *P < 0.05, **P < 0.01, ***P < 0.001, two-tailed Student’s *t*-test

Since cold resulted in increased fat deposition in muscle, we wanted to investigate its impact on intramuscular lipid composition. We applied mass-spectrometry-based lipidomic analysis to analyze the distribution and alteration of the lipid classes. With the orthogonal partial least-squares discrimination analysis (OPLS-DA) model, our analysis showed the clear separation of two classes (Additional file 2: Fig. S2A). We detected over 888 different lipid species in skeletal muscle, consisting of 248 TGs, 150 phosphatidylcholines (PCs), 126 phosphatidylethanolamines (PEs), and other lipid classes (Additional file 2: Fig. S2B). The detected lipid classes and their abbreviations are shown in Additional file 2: Fig. S2C. Our data showed that the overall lipid composition of muscle was significantly altered after cold exposure (Fig. 2A). Approximately 28% of total identified lipid species were significantly changed during cold environment, with 215 lipid species increased and 32 decreased (Fig. 2A), suggesting its potential contribution to muscle lipid remodeling. Quantification results based on lipid groups showed that the total lipid abundance of sphingolipids decreased significantly, but the abundance of glycerolipids, glycerophospholipids, fatty acyls, and saccharolipids remained unchanged after cold exposure (Additional file 2: Fig. S2D). Differential analysis of lipid class indicated that cold-treated muscle had elevated the abundance of TG, lysophosphatidylcholine (LPC), and lysophosphatidylethanolamine (LPE), showing 2.4-fold, 1.3-fold, and 1.8-fold increases over RT muscle, respectively (Fig. 2B). However, the sphingomyelin (SM) content of muscle was significantly decreased (Fig. 2B). We visualized all of the significantly changed lipid species by the bubble map and found the abundance of TG subclasses increased dramatically (Fig. 2C). To further probe individual lipid species that were altered by cold, we utilized the volcano plot to show changes in lipid subclasses of TG, LPC, LPE and SM (Fig. 2D). We next analyzed the composition of medium-and long-chain fatty acids in skeletal muscle and found that C16:1, C20:3N6, C20:4N6, C22:6N3, C14:1, C20:1, C18:3N3, and C20:5N3 were significantly increased after cold exposure (Additional file 2: Fig. S2E). In addition, cold-treated muscle showed a marked increase in the content of total fatty acid compared to RT muscle (Fig. 2E). Among these fatty acids, cold significantly increased the intensity of PUFAs, without affecting SFAs and MUFAs (Fig. 2F). Notably, cold exposure dramatically increased the level of n3-PUFA lipids, including DHA (C22:6N3) and EPA (C20:5N3) (Fig. 2G; Additional file 2: Fig. S2E), which are thought to improve muscle function and prevent muscle atrophy by enhanced anti-inflammatory responses as well as prevention of sarcopenia [27, 28]. Surprisingly, we also found a dramatic increase in the contents of total n6-PUFAs (Fig. 2G), which are reported to have some adverse impacts on human health and muscle function [20]. What is more, growing evidence suggests decreased n6 to n3 ratio is associated with increased metabolic disorders risk in type 2 diabetes patients [29]. We thus calculated the n6-PUFA/n3-PUFA ratios and found that the n6-PUFA/n3-PUFA ratio was significantly decreased by cold exposure (Fig. 2G). Overall, these findings suggest that cold exposure induced considerable alterations in the composition and content of lipid species in skeletal muscle.

### Fatty acyl chains associated with TG undergo dynamic change transitions

**Fig. 3:**
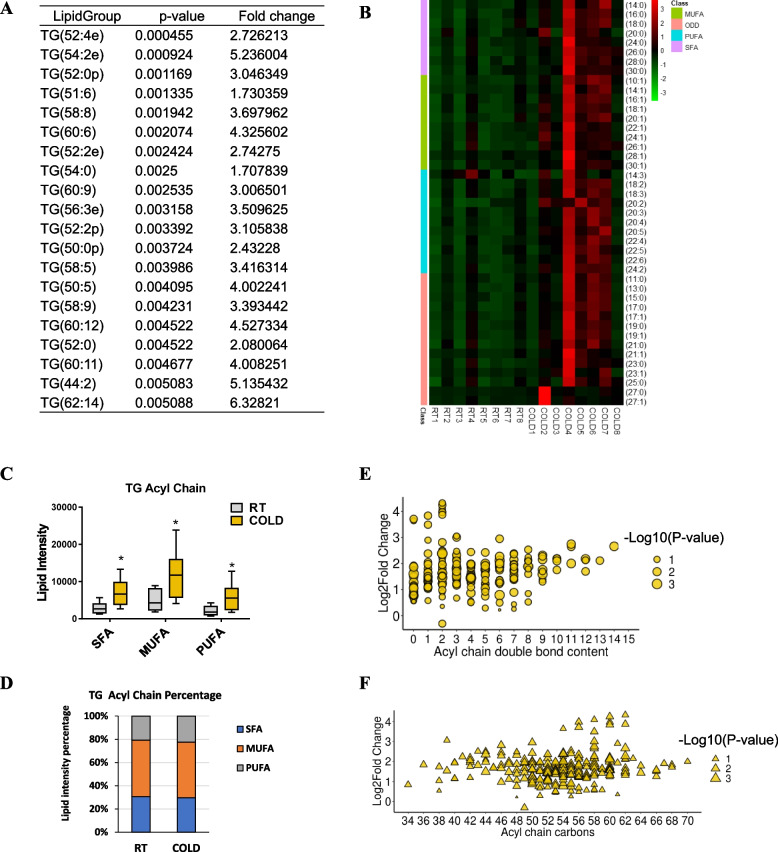
Cold-mediated changes in TG composition and fatty acyl chains in TA. **A** The top 20 TGs that change with cold exposure according to the *P* value (*n* = 8). **B** Heatmap showing the total intensity of individual fatty acyl chains of SFA, MUFA, PUFA, and ODD associated with TGs in the TA muscle of the RT and COLD group mice (*n* = 8). **C**, **D** Lipid intensity and percentage of individual fatty acyl chains of SFA, MUFA, or PUFA associated with TGs in the TA from the RT and cold-treated mice (*n* = 8). **E**, **F** Significantly changed TG species with acyl chain double bond and carbons numbers in TA from the RT and cold-treated mice (*n* = 8). Error bars represent s.e.m. *P < 0.05, **P < 0.01, ***P < 0.001, two-tailed Student’s *t*-test

Significant changes in muscle TG class prompted us to further determine the impact of cold exposure on the individual fatty-acyl-chain composition associated with TG lipid subclasses. Analysis of TG species revealed dramatic changes in the composition of the species (Additional file 3). We examined the top 20 species during cold conditions according to the *P* values (Fig. 3A). Notably, all species were significantly increased in muscle by cold exposure (Fig. 3A). Among them, TG species containing monounsaturated or polyunsaturated fatty acyl chains, e.g., TG (51:6), TG (52:4), TG (58:5), TG (58:9), and TG (58:8), showed the most dramatic relative increase in skeletal muscle upon cold exposure (Fig. 3A). Analysis of the individual fatty-acyl-chain composition indicated that most of the fatty acyl chains in TG were significantly increased by cold exposure (Fig. 3B; Additional file 3). In addition, the contents of most odd-number fatty acids showed a marked increase in the TG pool of skeletal muscle by cold exposure (Fig. 3B; Additional file 3). Interestingly, we also analyzed the composition of medium- and long-chain fatty acids in skeletal muscle and found that cold exposure could result in 3.5-fold, 5.0-fold, and 2.4-fold increases in the contents of DHA (C22:6), EPA (C20:5), and DPA (C22:5), respectively (Fig. 3B; Additional file 3), which is widely believed to be strongly beneficial to human health [30]. Consistently, significant increases in the total lipid density of SFAs, MUFAs, and PUFAs associated with TG acyl chains were observed in the cold exposure group (Fig. 3C). However, the total percentage of SFA, MUFA, or PUFA associated with TG remained unchanged in cold-treated skeletal muscle (Fig. 3D). In parallel, we evaluated the carbon numbers and double-bond content in TG, which has been reported to reflect the degree of unsaturation of very long chain fatty acid and human health [31]. Cold-induced skeletal muscle displayed the most markedly elevated levels of TG species with relatively lower double bond numbers (1~2 double bonds) and relatively higher acyl chain carbon numbers (58~62 carbons) (Fig. 3E, F). These findings demonstrated that the fatty acyl chains associated with TG undergo dynamic change upon cold exposure.

### Cold exposure caused transcriptional changes in skeletal muscle

**Fig. 4:**
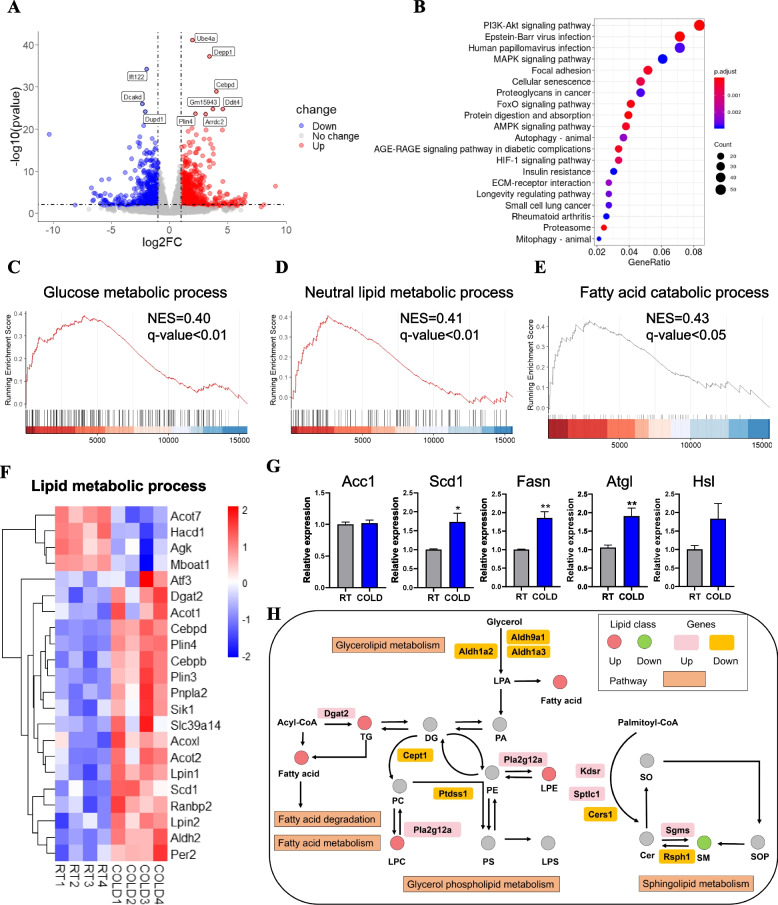
Short-term cold exposure induces transcriptome program alterations. **A** Volcano diagram of differentially expressed genes in TA from the RT and COLD-treated mice (*n* = 4). The transverse and vertical dotted lines indicate the cutoff value for differential expression (padj < 0.05 and Abs (Log2 fold change) > 1). Gray, red, and blue plots indicate no significant difference, increased differentially expressed, and decreased differentially expressed genes, respectively. **B** KEGG pathway analysis showing the enrichment of functional categories (*n* = 4). **C**–**E** Gene set enrichment analysis showing significant enrichment in fatty acid catabolic process, neutral lipid metabolic process, and glucose metabolic process from the RT and cold-treated mice (*n* = 4). **F** Heatmap showing the differentially expressed genes related to lipid metabolic processes (*n* = 4). **G** qPCR analysis of *Fasn*, *Scd1*, *Acc1*, *Atgl*, and *Hsl* in TA muscle during the cold challenge (*n* = 4). **H** Glycerolipid, glycerophospholipid, and sphingolipid metabolism from KEGG, with indications of lipid classes and genes significantly regulated in muscle by cold exposure (*n* = 4). Error bars represent s.e.m. *P < 0.05, **P < 0.01, ***P < 0.001, two-tailed Student’s *t*-test

To explore the underlying mechanism of dynamic change of skeletal muscle lipidome upon cold exposure, we applied RNA sequencing (RNA-seq) to map the transcriptional changes in skeletal muscle in response to short-term cold exposure. We found a total of 1653 differentially expressed genes, of which 793 were increased and 860 were decreased (Fig. 4A). Heatmap cluster analysis nicely distinguished the expression pattern of differentially expressed genes in the RT and cold-treated muscles (Additional file 4: Fig. S3A).

To unravel the function of differentially expressed genes, we performed Gene Ontology (GO) and Kyoto Encyclopedia of Genes and Genomes (KEGG) pathway analyses using clusterProfiler software. GO enrichment analysis of the elevated genes by cold stress revealed the pronounced changes in response to nutrient levels, cellular response to nutrient levels, response to extracellular stimulus, autophagy, and process utilizing autophagic mechanism (Additional file 4: Fig. S3B). In contrast, the enriched biological processes that were reduced in the cold-treated muscle included external encapsulating structure organization, extracellular structure organization, extracellular matrix organization, collagen fibril organization, regulation of neurogenesis, and synapse organization (Additional file 4: Fig. S3B), which play a multifunctional role in skeletal muscle development and muscle cell behavior. These results suggest that cold exposure altered the expression of genes involved in metabolic progression. Similarly, KEGG pathway analysis revealed a significant enrichment in several major metabolic pathways, including the glucose metabolic process, PI3K-Akt signaling pathway, MAPK signaling pathway, AMPK singling pathway, and insulin resistance (Fig. 4B; Additional file 4: Fig. S3C-G). We further determined the expression pattern of significantly changed genes involved in these pathways (Additional file 4: Fig. S3C-G). Furthermore, we performed gene set enrichment analysis (GSEA) to identify the biological processes of cold-treated muscle. Notably, the GSEA results showed that the cold-treated muscle exhibited significant changes in the fatty acid catabolic process, neutral lipid metabolic process, and glucose metabolic process (Fig. 4C–E). These results indicated that cold exposure could lead to changes in multiple signaling pathways in vivo. We further generated heatmaps to show the expression of genes enriched in glucose and lipid metabolic processes and found that genes related to glucose metabolism had strongly upregulated expression (Fig. 4F; Additional file 4: Fig. S3C). A series of genes associated with lipid metabolism were differentially expressed in cold-treated muscle (Fig. 4F). Among them, the expression of genes related to fatty acid and TG synthesis, including *Scd1*, *Dgat2*, *Lpin2*, *Cebpβ*, *Aldh2*, *Plin3*, and *Plin4*, was highly expressed in cold-treated muscle (Fig. 4F), suggesting that cold promoted fatty acid synthesis in skeletal muscle. To validate the lipid synthesis in muscle upon cold stress, we performed the qPCR analysis to confirm significantly increased expression levels of key muscle lipogenesis regulators in the cold-treated muscle, as revealed by the higher level of *Scd1* and *Fasn* (Fig. 4G), which play an essential role in fatty acid biogenesis and lipid deposition [32, 33]. Moreover, *Pnpla2 (Atgl)*, a key component regulating TG hydrolysis, was highly expressed in the cold-treated muscle (Fig. 4G), suggesting cold exposure increased TG breakdown in muscle.

To determine the role of cold exposure in regulating lipid metabolism and remodeling in muscle, we integrated lipidomics with transcriptomics to provide a comprehensive overview of the cold effect on muscle based on KEGG (Fig. 4H). These differentially expressed genes participated in muscle lipid remodeling through complex networks. Based on the above results, the increased level of TGs might be caused by activated intramuscular fatty acid synthesis and TG hydrolysis.

### Cold exposure induced mitophagy and mitochondrial dysfunction

**Fig. 5:**
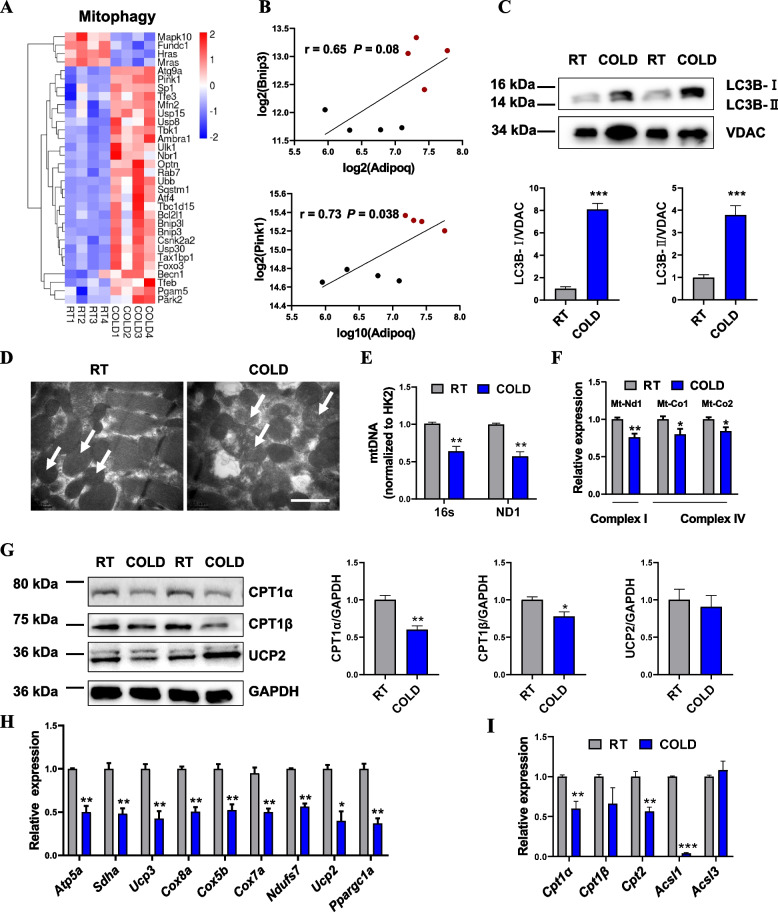
Cold exposure induced mitophagy and mitochondrial dysfunction. **A** Heatmap showing the differentially expressed genes related to mitophagy after cold exposure (*n* = 4). **B** Correlation analysis of mRNA expression of *Adipoq* with *Bnip3* or *Pink1* in TA muscle (*n* = 4). *r*, Pearson’s correlation coefficient. **C** Immunoblot analysis of mitochondrial-associated LC3B levels in mitochondrial-enriched fraction from the RT and COLD-treated mice, VDAC was used as a loading control. **D** Representative TEM images of TA muscle from the RT and COLD-treated mice (*n* = 4). **E** mtDNA levels in TA from the RT and COLD-treated mice (*n* = 6). **F** qPCR analysis of mitochondrial respiratory chain subunit genes in TA muscle. Mt-ND1, mitochondrially encoded NADH dehydrogenase 1; Mt-Co1, mitochondrial cytochrome c oxidase 2 gene; Mt-Co2, mitochondrial cytochrome c oxidase 2 gene (*n* = 4). **G** Western blot of CPT1α, CPT1β, and UCP2 protein expression in TA muscle from the RT and COLD-treated mice, GAPDH was used as a loading control. **H** qPCR analysis of genes related to mitochondrial biogenesis in TA from the RT and COLD-treated mice (*n* = 4). **I** qPCR analysis of genes related to fatty acid oxidation in TA from the RT and COLD-treated mice (*n* = 4). Error bars represent s.e.m. *P < 0.05, **P < 0.01, ***P < 0.001, two-tailed Student’s *t*-test

Autophagy has been reported to play an important role in modulating lipid metabolism in multiple tissues. Notably, we also found that genes involved in autophagy and mitophagy processes were significantly changed upon cold exposure based on GSEA analysis (Additional file 5: Fig. S4A-C). RNA-seq and GSEA results prompted us to investigate the potential role of autophagy in cold-induced fatty acid metabolism. Heatmap cluster analysis showed differentially expressed genes related to autophagy and mitophagy that were highly induced by cold exposure (Fig. 5A; Additional file 5: Fig. S4D). We compared the expression of mitophagy-related genes in the cold-treated and control groups. As shown by the heatmap, the expression of damaged mitochondrial priming adapters (*Pink1* and *Park2*) and several mitophagy receptors (such as *Bnip3l*, *Bnip3*, *Ambra1*) was significantly induced by cold exposure (Fig. 5A). The expression of *Tbk1*, encoding tank-binding kinase 1, which promotes mitophagy, was also significantly increased after cold exposure (Fig. 5A). Expression of *Adipoq* mRNA in the muscle was significantly increased after cold exposure and showed a positive correlation with *Pink1* mRNA expression (Fig. 5B), suggesting increased muscle TG positively correlated with activated autophagy.

To examine the effect of cold exposure on autophagy in skeletal muscle in vivo, we first examined the biochemical hallmarks of autophagy in the TA muscle during a cold challenge. Notably, autophagy and mitophagy were sensitive to acute cold exposure (Additional file 5: Fig. S4D-F). LC3 is a soluble protein which is employed for quantifying autophagy in vitro and in vivo. The content of LC3-II is proportional to the degree of autophagy, and the ratio of LC3B-II/I can reflect the level of autophagy [34]. Western blot analysis revealed slightly increased LC3-I to LC3-II conversion and BNIP3 protein levels in total muscle lysate (Additional file 5: Fig. S4E). qPCR analysis showed that autophagy-related genes did not change significantly after cold exposure, but mRNA expression levels related to mitophagy were elevated (Additional file 5: Fig. S4F). Similar results were observed in mitochondrial-enriched samples prepared from the cold-treated muscle compared to the controls (Fig. 5C). Consistent with the enhanced mitophagy shown by analysis of mRNA and protein, TEM images revealed the presence of numerous mitophagosomes and damaged mitochondrial structure in the cold-challenged mouse muscle (Fig. 5D). In addition, the qPCR analysis revealed marked decrease in mtDNA levels in the cold-treated muscle compared with the controls (Fig. 5E). In parallel, we observed significantly decreased levels of mitochondrially encoded NADH dehydrogenase 1 (*mt-Nd1*), mitochondrially encoded DNA cytochrome c oxidase I (*mt-Co1*), and cytochrome c oxidase II (*mt-Co2*) (Fig. 5F). Together, these results suggest enhanced LC3-mediated mitophagy was observed in muscle during acute cold exposure.

Although mitochondrial metabolism serves only a secondary role in supplying energy for thermogenesis in skeletal muscle, it has been reported that the intermyofibrillar mitochondria in skeletal muscle become more elongated and tubular upon adaptation to severe cold [35]. To reveal the role of mitochondria in skeletal muscle during cold acclimatization, we observed inhibition of mitochondrial biogenesis-related gene expression after cold treatment in muscle. Similar to our in vivo observations after cold exposure, we found that the levels of mitochondrial oxidative phosphorylation and UCP2 in skeletal muscle were slightly decreased by cold exposure (Fig. 5G; Additional file 5: Fig. S4G, H). In addition, fatty acid oxidation-related protein levels, including CPT1α and CPT1β, were significantly decreased after acute cold stress (Fig. 5G). Consistent with our findings, cold-stressed mice had a decreased oxidative capacity and impaired mitochondrial function in the TA muscle, as a result of reduced expression of genes related to fatty acid oxidation and mitochondrial biogenesis (Fig. 5H, I). These results suggested that short-term cold treatment might cause damage to mitochondria and induce mitophagy in skeletal muscle, resulting in lipid accumulation in the cold-treated skeletal muscle.

### Cold exposure activated the HIF-1α signaling pathway to regulate triglyceride deposition and promote mitophagy in skeletal muscle

**Fig. 6:**
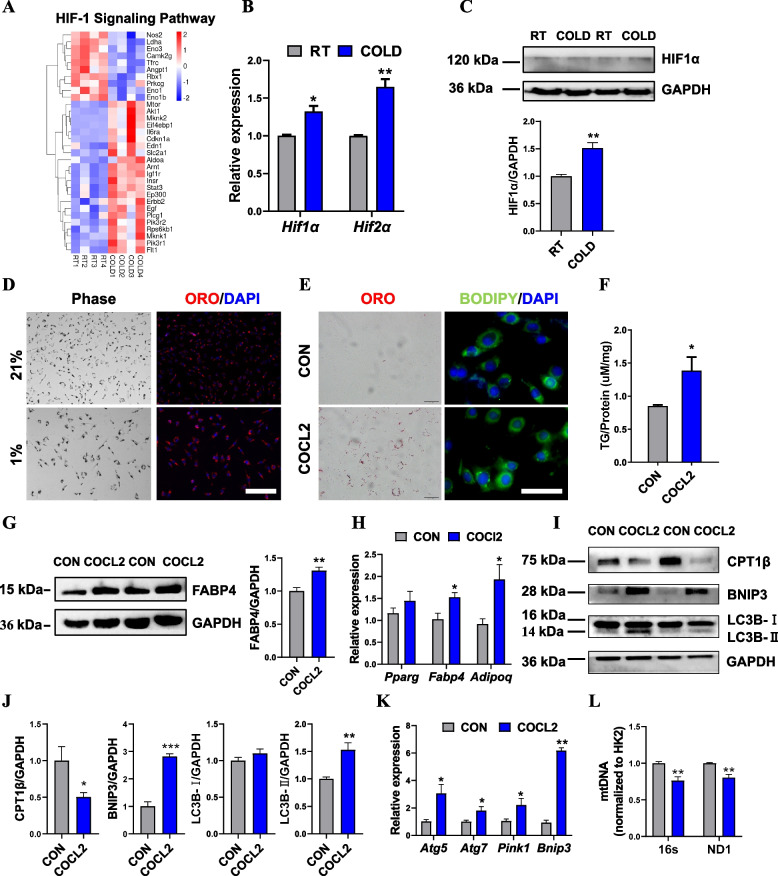
Activated HIF1a signaling regulates fat deposition and mitophagy in response to the cold challenge. **A** Heatmap showing the differentially expressed genes related to HIF-1α signaling (*n* = 4). **B** mRNA expression of *Hif-1α* and *Hif-2α* in TA from the RT and COLD-treated mice (*n* = 4). **C** Western blot of HIF-1α protein expression in TA from the RT and COLD-treated mice, GAPDH was used as a loading control. **D** Oil red O staining in primary myoblasts from normoxia (21%) and hypoxia (1%) for 48 h. **E** Oil red O and BODIPY staining from the control (CON) and COCL2-treated C2C12 myoblasts for 24h. **F** TG contents from CON and COCL2-treated C2C12 myoblasts for 24h (*n* = 6). **G** Immunoblot analysis of FABP4 levels from the CON and COCL2-treated C2C12 myoblasts for 24h. **H** qPCR analysis of genes related to lipid metabolism from the CON and COCL2-treated C2C12 myoblasts for 24 h (*n* = 4). **I**, **J** Immunoblot analysis of LC3B, CPT1B, and BNIP3 levels from the CON and COCL2-treated C2C12 myoblasts for 24 h. **K** qPCR analysis of genes related to autophagy and mitophagy from the CON and COCL2-treated C2C12 myoblasts for 24h (*n* = 4). **L** mtDNA levels in the CON and COCL2-treated C2C12 myoblasts for 24h (*n* = 6). Error bars represent s.e.m. *P < 0.05, **P < 0.01, ***P < 0.001, two-tailed Student’s *t*-test

**Fig. 7:**
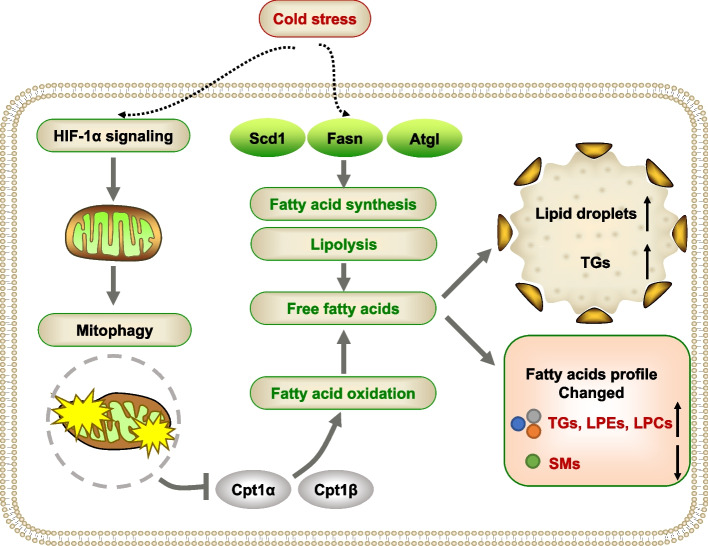
A working model of short-term cold exposure in regulating lipid metabolism in skeletal muscle. Short-term cold exposure leads to increased intramuscular lipid droplet deposition in skeletal muscles by mobilizing the body’s metabolism. Cold promotes the process of lipolysis and fatty acid synthesis in the body to generate free fatty acids to store energy. Consequently, the cold challenge induces muscle lipid remodeling and significant changes in lipid composition, especially triglycerides. Transcriptome results show markedly changed genes involved in fatty acid metabolism and mitophagy. Notably, cold exposure leads to impaired mitochondrial oxidation and enhanced mitophagy process, which is modulated by activated HIF1a signaling

To explore the cold exposure-induced signaling pathways regulating mitochondrial dysfunction and autophagy, as well as lipid metabolism in skeletal muscle, we focused on pathways significantly enriched based on RNA-seq results. Among these pathways, HIF-1α signaling pathway has been reported to participate in the regulation of lipid metabolism in a variety of tissues [36]. Furthermore, HIF-1α signaling is also closely associated with mitophagy and adaptive responses induced by cold exposure [37, 38]. Therefore, we first determined the changes in gene expression profiles upon cold exposure based on RNA-seq results. The heatmap results showed that several HIF-1α signaling pathway-related genes (*Mtor*, *Erbb2*, *Plcg1*, *Igf1r*, and *Insr*) were increased significantly (Fig. 6A). Consistently, qPCR and western blot analysis showed that cold exposure enhanced *HIF-1α* mRNA and protein expression in skeletal muscle (Fig. 6B, C). It has reported that HIF-1α drives lipid deposition in clear cell renal cell carcinoma by repressing CPT1α, subsequently reducing fatty acid transport into the mitochondria and forcing fatty acids to be stored as lipid droplets [39]. We hypothesized that the increased TG contents in the cold-treated skeletal muscle might be a result of impaired β-oxidation caused by cold-induced hypoxia and regulated by the HIF-1α signaling pathway. Oil red O staining showed that hypoxia (1% O_2_) dramatically induced lipid accumulation in primary myoblasts (Fig. 6D). We next constructed a hypoxic cell model using COCl_2_ and Oil Red O staining showed that COCl_2_ strongly induced lipid accumulation in C2C12 myoblasts (Fig. 6E). Consistently, we also found increased TG levels and elevated gene and protein expression involved in fat synthesis and adipocyte differentiation in the COCl_2_-treated C2C12 myoblasts (Fig. 6F–H). We next determined the role of HIF-1α signaling in the regulation of autophagy and mitophagy. qPCR and western blot results showed that enhanced HIF-1α significantly promoted intracellular autophagy due to increased protein expression of BNIP3 and LC3B (Fig. 6I, J). In parallel, the mRNA levels related to autophagy and mitophagy, including *Atg5*, *Atg7*, *Pink1*, and *Bnip3*, were elevated significantly (Fig. 6K). Consistently, the qPCR analysis revealed marked decreases in mtDNA levels in the COCl2-treated C2C12 myoblasts compared with the controls (Fig. 6L). Together, these results suggested that the activated HIF-1α signaling pathway may contribute to elevated TG levels and mitochondrial dysfunction in skeletal muscle after cold exposure (Fig. 7).

## Discussion

In the current study, we found that short-term cold exposure significantly increased intramuscular fat deposition. Moreover, we applied mass-spectrometry-based lipidomics and RNA-seq to extend our understanding of the alteration of lipid metabolism and its pathways in skeletal muscle in response to short-term cold exposure. Our results showed that cold exposure induced significant changes in the composition of specific lipid species, especially TG species. The length of acyl chains associated with TG and the composition of medium- and long-chain fatty acids were also influenced by cold treatment. RNA-seq results indicated that a cold challenge induced gene programs involved in the regulation of glucose and fatty acid metabolism. In addition, our results revealed that LC3-mediated autophagy and mitophagy induced by the HIF-1α signaling pathway may play a key role in excessive lipid accumulation in cold-treated skeletal muscle.

Cold stress can activate thermogenic processes and mobilize metabolic remodeling and multiple physiological changes in vivo [40–42]. Our results showed that acute cold exposure increased intramuscular fat deposition, which was accompanied by changes in glucose and lipid metabolic process in muscle. This finding was consistent with previous reports that cold exposure causes fiber type-specific responses in glucose and lipid metabolism in rat skeletal muscles [16]. We found that the mRNA levels of *Fabp4*, *Pparg*, and *Plin1* were significantly elevated, confirming the cold-induced regulation of lipid metabolism. Research indicates that acute exercise increases TG synthesis in skeletal muscle and prevents fatty acid-induced insulin resistance [43]. This result may also reveal that increased TG in muscle is due to partitioning more fatty acids toward TG synthesis within muscle, reducing the accumulation of fatty acid metabolites in cold environments. Skeletal muscle fatty acid composition is tightly linked to lipid metabolism. Previous studies revealed extensive changes in lipid compositions and lipid metabolism related-pathways in BAT and iWAT in response to short-term cold exposure [20, 44]. Thus, we further evaluated the effect of cold exposure on the fatty acid profile of skeletal muscle. We found that the compositions of lipid classes were remodeled selectively by short-term cold adaptation in skeletal muscle, especially in terms of the total contents of TGs. This finding further demonstrated that the cold challenge induced increased fat deposition in muscle. Notably, our previous results showed that short-term cold exposure altered fatty acid composition in fat-infiltrated muscles through directly affecting lipid metabolic pathways including the PI3K-AKT and MAPK signaling pathways [45]. Several lipids have been recently identified as signaling molecules (lipokines) involved in the regulation of systemic metabolism and insulin sensitivity in the body [46, 47]. Lipidomic results showed that cold exposure caused significant changes in the composition and content of a class of lipids. Several pieces of evidence suggest that aged muscle exhibits reduced TG and PE and increased SM content [48], whereas cold induced intramuscular lipid remodeling and the changes in fatty acid composition, especially reduced SM content and increased LPE content, which may be useful for preventing aging and metabolic diseases. In addition, a key finding was that short-term cold exposure was sufficient to cause a significant increase in the levels of n3-PUFA lipids, including DHA (C22:6N3) and EPA (C20:5N3), which were copreventative and cotherapeutic for human health [30]. These results indicate lipid remodeling induced by cold may play a beneficial role in the body.

To identify the key signaling pathways involved in cold exposure-induced changes in lipid metabolism, we performed RNA-seq analysis and found a large number of differentially expressed genes related to glucose and lipid metabolism. This finding was consistent with our lipidome results. Specifically, the expression levels of a series of differentially expressed genes related to fatty acid synthesis, such as *Scd1* and *Fasn*, were significantly upregulated. In addition, enhanced TG lipolysis in muscle was observed, as evidenced by increased expression levels of *Atgl* and *Hsl*. Substantial evidence shows that cold-induced adrenergic signaling also induces intracellular TG lipolysis via the activation of triglyceride lipase (ATGL) and hormone-sensitive lipase (HSL) in adipose tissue [49]. Hence, cold exposure-mediated non-shivering thermogenesis could also lead to enhanced breakdown in muscle.

Skeletal muscle contributes to regulatory heat production, which involves both shivering and non-shivering processes. In particular, uncoupling in skeletal muscle mitochondria has been suggested as a source of heat production [42]. The switch in metabolism from carbohydrate to lipid catabolism was observed in cold-acclimated hind limb muscles, which was accompanied by an increased total mitochondrial complement and ATP production capacity in the hind limb muscles of cold-acclimated individuals [50]. This finding was consistent with our results indicating that cold affected muscle lipid metabolism and mitochondrial function. Different studies have indicated the effect of cold exposure on energy metabolism in skeletal muscle under different experimental conditions. It has recently been demonstrated that cold acclimation (several weeks at 4°C), which is mediated mostly by non-shivering thermogenesis, enhanced mitochondrial yield and elaborate crista structure [40] and increased the fatty acid oxidative capacity [50]. Another study showed that 7-day cold-challenged skeletal muscle is activated but demonstrated no changes in mitochondrial biogenesis and autophagy-related proteins [51]. In our study, impaired mitochondrial function and enhanced mitophagy were observed in cold-treated muscle. Our data highlighted the crucial importance of mitophagy in lipid metabolism and mitochondrial homeostasis. Mitophagy is responsible for maintaining the quantity and quality of mitochondria, as well as normal cellular and organismal metabolism. During an acute cold environment, enhanced mitophagy selectively removed superfluous and damaged mitochondria, and it was coordinated with mitochondrial biogenesis to reduce the fatty acid oxidative capacity.

Upon exposure to cold stress, the oxygen consumption of animals increases by means of shivering and non-shivering thermogenesis [52]. It was assumed that an increased demand for oxygen during cold exposure could induce local hypoxia [53]. Mitophagy is induced to decrease mitochondrial quantity, thereby adapting cellular metabolism to anaerobic conditions when exposed to hypoxia [54]. In this study, short-term cold exposure activated the HIF-1α signaling pathway in skeletal muscle, which may disrupt mitochondrial homeostasis in cells. Previous studies demonstrated that hypoxia induced mitochondrial autophagy through a HIF-1α-dependent adaptive metabolic response, which required the constitutive expression of *Bnip3*, *Beclin-1*, and *Atg5* [55]. Mechanistic studies further revealed that hypoxia induced dephosphorylation of FUNDC1 and enhanced its interaction with LC3 for selective mitophagy in mammalian cells [56]. Our RNA-seq data revealed that short-term cold exposure induced significant enrichment in the mitophagy pathway, the mRNA levels of *Bnip3* and *Beclin-1* were significantly elevated, and the mRNA levels of *Fundc1* were significantly decreased by cold treatment. In addition, hypoxia has been shown to rapidly increase the level of plasma triglycerides in mice by decreasing plasma lipoprotein clearance [57]. The rate-limiting component of mitochondrial fatty acid transport, CPT1α, is repressed by hypoxia, impairing fatty acid metabolizing enzymes, reducing fatty acid transport into the mitochondria, and forcing fatty acids to lipid droplets for storage in cancer cells [39, 58]. We observed that the expression of CPT1α was inhibited by cold exposure and that hypoxia could induce lipid accumulation in muscle cells in vitro. This evidence combined with our results mentioned above supported the hypothesis that the oxygen deficit induced by shivering and non-shivering thermogenesis by short-term cold exposure might activate the HIF-1α signaling pathway, subsequently impairing mitochondrial function by inhibiting CPT1α and triggering mitophagy, which ultimately resulted in lipid accumulation. However, additional studies are required to determine how cold exposure induced the HIF-1α signaling pathway.

In summary, our results revealed the crucial role of short-term cold exposure in regulating lipid metabolism and lipid remodeling in skeletal muscle. Enhanced mitophagy further suggests its contribution to the maintenance of metabolic homeostasis, which was regulated by HIF-1α signaling.

## Conclusions

In conclusion, our results show that short-term cold exposure leads to increased lipid deposition which is associated with lipid remodeling in the muscle upon cold exposure. Transcriptome results confirmed the significant changes in gene expression related to glucose and lipid metabolism. Mechanistically, mitophagy may play a critical role in mobilizing thermogenesis and regulating metabolism in skeletal muscle, which is precisely regulated by HIF-1α signaling.

## Methods

### Animals

All animal experiments were performed according to protocols approved by the Zhejiang University Animal Care and Use Committee. Ten-week-old male C57BL6/J mice were purchased and housed under controlled temperature at 22 °C and a 12-h light/12 h dark cycle, with free access to a standard chow diet and water. For acute cold exposure experiments, six mice were placed at either 23°C or 4 °C for 3 days on a 12-h light–dark cycle and then killed for serum and tissue collection. In all experiments, TA, SOL, EDL, GAS, iWAT, and BAT were dissected, frozen immediately in liquid nitrogen, and stored at −80 °C.

### Cell culture and BODIPY staining

C2C12 myoblasts were maintained in 5% CO2 at 37°C and grown in DMEM containing 10% fetal bovine serum, penicillin (100 U/ml), and streptomycin (100 μg/ml). C2C12 myoblasts were treated with PBS or 200 μM COCL2 (Sigma-Aldrich). For BODIPY staining, cells were fixed with 4% paraformaldehyde for 15 min and incubated with 0.5 nM BODIPY FL (Invitrogen) for 10 min. Then, lipid droplets were observed by fluorescence microscopy.

### Hematoxylin-eosin (H&E) staining

TA muscle, brown and white adipose tissues from the RT and cold-treated mice were fixed in 4% paraformaldehyde for 2 h at room temperature. Then, the tissues were embedded into paraffin, blocked, and cut at 10 μm for H&E staining. The sections were deparaffinized, rehydrated, and stained with hematoxylin for 15 min. Sections were then rinsed in running tap water, stained with eosin for 3–5 min, dehydrated, mounted, and captured.

### Transmission Electron Microscopy (TEM)

TEM assays were performed as described. Electron photomicrographs were taken from cell ultrastructures under a transmission electron microscope (Hitachi, H-7650).

### Gene expression and mitochondrial DNA content

Total RNA extraction and quantitative real-time PCR were performed as previously described [20]. Briefly, total RNA was extracted from tissues and cells using TRIzol Reagent (Thermo Fisher Scientific). One microgram of RNA was reverse transcribed with a high-capacity cDNA synthesis kit. Real-time PCR was conducted with an Applied Biosystems StepOnePlus™ Real-Time PCR System using SYBR Green Master Mix (Roche). The relative abundance of mRNA was calculated after normalization to 18S ribosomal RNA expression. Total DNA was extracted from isolated TA muscle using the Tissue Mitochondria Isolation Kit (Beyotime Biotechnology). Relative mtDNA content was determined as the ratio of the copy numbers from the mtDNA-encoded gene (ND1 and 16S) to the nuclear DNA-encoded gene (HK2). A list of gene-specific primers is available in Additional file 6.

### Protein extraction and western blotting

Total protein extraction and western blotting were performed as previously described [20]. Briefly, homogenized tissues and cells were lysed in RIPA buffer, and total protein concentrations were determined using Pierce BCA Protein Assay Reagent (Thermo Fisher Scientific). Equivalent protein quantities were subjected to SDS-PAGE, transferred to a polyvinylidene fluoride membrane (Millipore Corporation), and blocked in 5% fat-free milk for 1 h at room temperature. The membranes were then probed with primary antibodies in 5% milk overnight at 4 °C, followed by the appropriate HRP-conjugated secondary antibodies. The primary antibodies were the following: rabbit anti-UCP1 (Abcam, Cat #: ab23841, Lot: GR3188478-15, RRID: AB_2213764) 1:2000, rabbit anti-FABP4 (HUABIO Biotechnology, Cat #: ET1703-98) 1:2000, mouse anti-GAPDH (HUABIO Biotechnology, Cat #: EM1101, Lot: HG0718, RRID: AB_2811078) 1:5000, rabbit anti-UCP2 (Proteintech, Cat# 11081-1-AP, RRID: AB_2213793) 1:1000, rabbit anti-CPT1B (GeneTex Cat# GTX117231, RRID:AB_10722705) 1:1000, rabbit anti-CPT1A (Abcam Cat# ab220789, RRID:AB_2847832), rabbit anti-LC3B (Cell Signaling Technology, Cat# 12741, RRID:AB_2617131) 1:1000, rabbit anti-VDAC (HUABIO Biotechnology, Cat# ET1601-20), and rabbit anti-BNIP3 (Thermo Fisher Scientific, Cat# PA5-22859, RRID:AB_11155377). The secondary antibodies were the following: anti-rabbit IgG 1:5000 (Prod #: 31460, Lot #: RB230194, RRID: AB_228341, Thermo Fisher Scientific) and anti-mouse IgG 1:5000 (Prod #: 31430, Lot #: RJ240410, RRID: AB_2040944, Thermo Fisher Scientific). Immunodetection was performed using an enhanced chemiluminescence western blotting substrate (Google Biotechnology) and detected with a ChemiScope3500 mini System.

### Immunofluorescence

Cultured C2C12 myoblasts were fixed in 4% paraformaldehyde and permeabilized for 10 min in 0.3% Triton X-100 in PBS. The samples were blocked in 10% goat serum for 1 h at room temperature. Primary antibodies were incubated in a blocking buffer at 4 °C overnight. Subsequently, the samples were washed with PBS and stained with the appropriate fluorescently labeled secondary antibodies (Alexa Fluor 488 or 594) for 1 h at room temperature. After washing with PBS, DAPI (Roche) was used to stain nuclei for 8 min. The primary antibodies used were as follows: rabbit anti-laminin (Sigma-Aldrich, Cat# L9393, RRID:AB_477163) 1:200.

### Lipidomic analysis

Lipid extraction and mass-spectrometry-based lipid detection were conducted as previously described [20]. Briefly, 200 μL cold water and 20 μL lipid standard internal mixture were added to TA muscle for lipid extraction, and then, the samples were homogenized on an MP homogenizer at 4°C. Afterwards, 800 μL cold methyl tert-butyl ether and 240 μL methanol were added to the samples and vortexed for 30 s, sonicated at 4°C for 20 min and stood for 30 min, then centrifuged (14,000 g for 15 min at 10 °C) to extract lipids. The upper organic layer was dried in a vacuum centrifuge. The dried samples were resuspended in 200 μL of isopropanol acetonitrile 9:1 (v/v) and used for lipidomic analysis.

For lipidomic analysis, lipid extracts were analyzed by LC-MS. LC-MS/MS analysis was performed on a Q Exactive plus mass spectrometer (Thermo Scientific) coupled to a UHPLC Nexera LC-30A (Shimadzu). In brief, lipids were separated on a Waters ACQUITY PREMIER CSH C18 Column (1.7 μm×2.1×100 mm) under the following chromatographic conditions: mobile phase A (acetonitrile: water = 6:4, v/v) and mobile phase B (acetonitrile: isopropanol = 1:9, v/v) at a flow rate of 300 μL/min and column oven temperature at 45 °C. MS detection was performed and analyzed with electrospray ionization (ESI) in positive and negative ion modes. Full-scan spectra were collected in mass-to-charge ratio (*m*/*z*) ranges of 200–1800 and 250–1800 for positive and negative ion modes, respectively. Lipid identification, peak extraction, peak alignment, and quantification were assessed with LipidSearch software version 4.1 (Thermo Scientific™). Differential abundance analysis was performed using a two-tailed Student’s *t*-test. The result of the lipidomic analysis is provided in Additional file 3.

### RNA-seq analysis

Total RNA was extracted from muscle tissue and treated with RNase-free DNase I to avoid contamination by genomic DNA. A total amount of 2 μg RNA per sample was used for the RNA sample preparation. The cDNA library construction and sequencing were performed by Sangon Biotech (Shanghai) Co., Ltd. (Shanghai, China). DESeq2 was used to conduct transcriptome data analysis between two samples. Differentially expressed genes were considered as if *q* value < 0.05 and Log2 FoldChange > 1. The results of RNA-seq analysis are provided in Additional file 7.

### Data analysis

All the statistical evaluations of lipidomic data described in this work were calculated from relative abundances. Experimental data are presented as the mean ± SEM. Comparisons were made by unpaired two-tailed Student’s *t* tests. Differences among groups were considered statistically significant at *P* < 0.05.

## Supplementary Information

**Additional file 1: Fig. S1.** Cold exposure for 3 days leads to IWAT browning and increased thermogenesis. (A-B) Cold exposure decreases the body weight and the mass of BAT, eWAT and iWAT (n = 8). (C) H&E staining of iWAT sections from control and cold-treated mice. (D) Western blots and quantitative analysis of UCP1 protein levels in IWAT. (E) Western blots and quantitative analysis of UCP1 protein levels in BAT. (F) mRNA of BAT- selective related genes in iWAT from control and cold-treated mice (n = 6). (G) mRNA of *Ucp1* genes in iWAT from control and cold-treated mice (n = 6). Error bars represent s.e.m. * P < 0.05, ** P < 0.01, *** P < 0.001, two-tailed Student’s t-test.

**Additional file 2: Fig. S2.** Cold exposure alters skeletal muscle lipid composition. (A) OPLS-DA scores plot. Blue and green symbols represent RT and COLD samples, respectively. (B) Composition of lipid classes that were considered for subsequent analysis in all of the samples detected by LC-MS. (C) Quantified lipid classes and their abbreviations. (D) The intensity of glycerolipids, glycerophospholipids, fatty acyls, sphingolipids and saccharolipids in the TA muscle of the RT and COLD group mice. (E) Heatmap showing the total intensity of individual fatty acyl chains altered in COLD vs RT muscle. Error bars represent s.e.m.* P < 0.05, ** P < 0.01, *** P < 0.001, two-tailed Student’s t-test.

**Additional file 3.** Lipidomic data of muscle after cold exposure.

**Additional file 4: Fig. S3.** Short-term cold exposure induces transcriptome programs alterations. (A) Heatmap showing the differentially expressed genes (padj < 0.05 & Abs (Log2 fold changes) > 1) in TA muscles from control and cold-treated mice (n = 4). Red and blue indicate upregulated differential and downregulated differential expression genes, respectively. (B) Gene Ontology analysis showing the enrichment of functional categories (n = 4). (C) Heatmap showing the differentially expressed genes related to glucose metabolic processes (n = 4). (D-G) Heatmap showing the differentially expressed genes related to PI3K-Akt signaling pathway, MAPK signaling pathway, AMPK signaling pathway and Insulin resistance (n = 4).

**Additional file 5: Fig. S4.** Short-term cold exposure induces transcriptome programs alterations. (A-C) Gene set enrichment analysis showing significant enrichment in macroautophagy, autophagy, and mitophagy from control and cold-treated mice. (D) Heatmap showing the differentially expressed genes related to autophagy (padj < 0.05 & Abs (Log2 fold changes) > 1) in TA muscles from control and cold-treated mice. Red and blue indicate upregulated differential and downregulated differential expression genes, respectively (n = 4). (E) Western blot of BNIP3 and LC3B proteins expression in TA muscles from RT and cold-treated mice, GAPDH as loading control. (F) mRNA expression of the autophagy (*Atg5* and *Atg7*) and mitophagy (*Pink1*, *Park2*, and *Bnip3*) genes in TA muscles from RT and cold-treated mice (n = 4). (G-H) Western blot and quantitative analysis of mitochondrial fractions showing oxidative phosphorylation protein in TA muscles from RT and cold-treated mice, GAPDH as loading control. Error bars represent s.e.m.* P < 0.05, ** P < 0.01, *** P < 0.001, two-tailed Student’s t-test.

**Additional file 6.** Primers for qPCR.

**Additional file 7.** RNA-seq data of muscle after cold exposure.

**Additional file 8.** Full scans of immunoblots.

**Additional file 9.** Individual data values.

## Data Availability

All data generated or analyzed during this study are included in this published article, its supplementary information files, and publicly available repositories. RNA sequencing and lipidomic data are available in Additional files 7 and 3, respectively. Raw RNA-seq data are available in the Sequence Read Archive database (PRJNA911412). Raw lipidomic data are available in the public MetaboLights database (MTBLS6723). The individual data values for Figs. 4, 5, and 6, as well as Additional file 4: Fig. S3 and Additional file 5: Fig. S4, are provided in Additional file 9.
