# Supplementary figures and images for: Cold exposure alters lipid metabolism of skeletal muscle through HIF-1α-induced mitophagy
Source: BMC Biol. 2023 Feb 8;21:27. doi: 10.1186/s12915-023-01514-4 (PMC9906913; doi:10.1186/s12915-023-01514-4)

Fig. S1

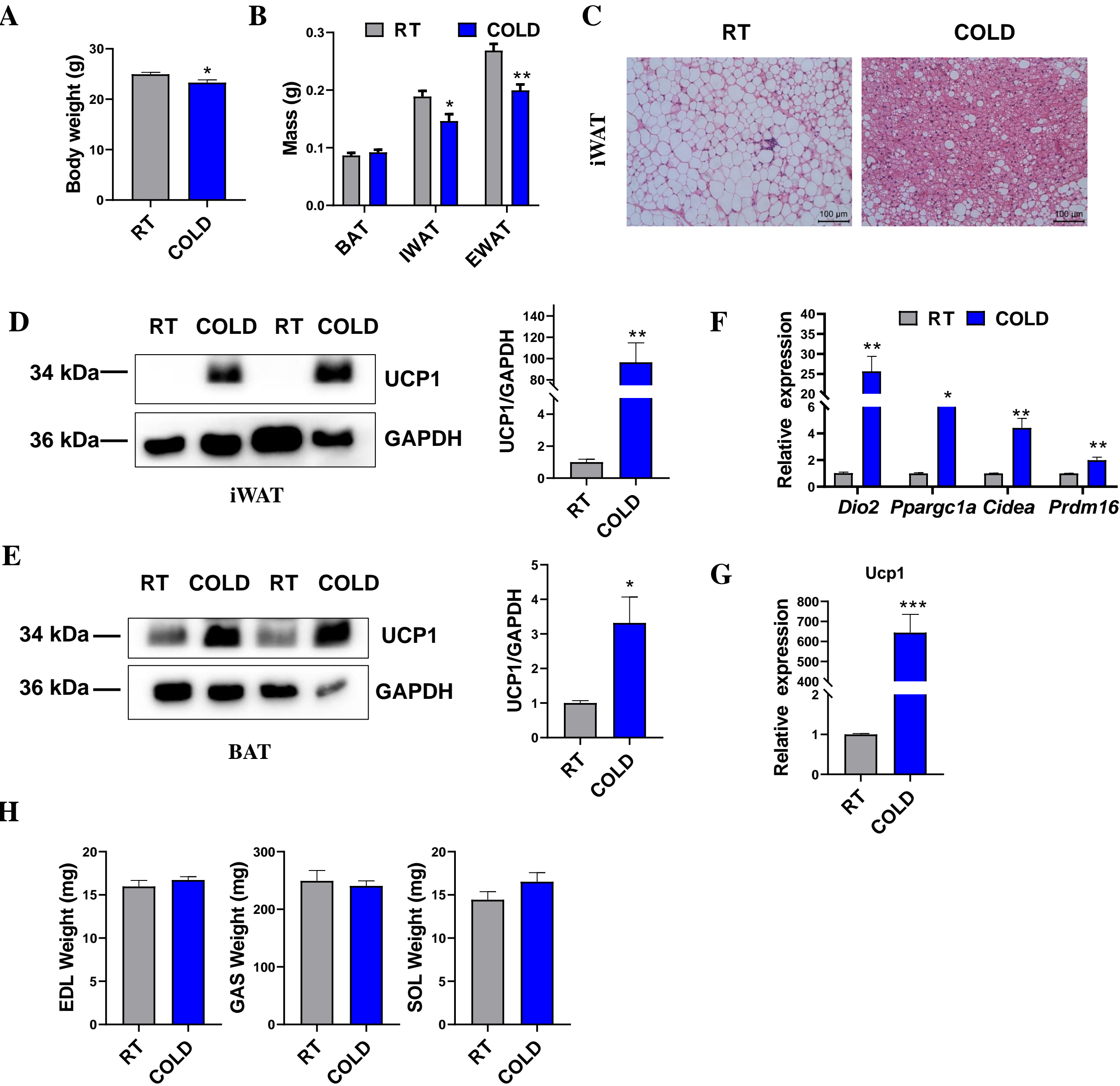

Supplement: Supplementary file 1 — Additional file 1: Fig. S1. Cold exposure for 3 days leads to IWAT browning and increased thermogenesis. (A-B) Cold exposure decreases the body weight and the mass of BAT, eWAT and iWAT (n = 8). (C) H&E staining of iWAT sections from control and cold-treated mice. (D) Western blots and quantitative analysis of UCP1 protein levels in IWAT. (E) Western blots and quantitative analysis of UCP1 protein levels in BAT. (F) mRNA of BAT- selective related genes in iWAT from control and cold-treated mice (n = 6). (G) mRNA of Ucp1 genes in iWAT from control and cold-treated mice (n = 6). Error bars represent s.e.m. * P < 0.05, ** P < 0.01, *** P < 0.001, two-tailed Student’s t-test. [file 12915_2023_1514_MOESM1_ESM.pdf]

Fig. S3

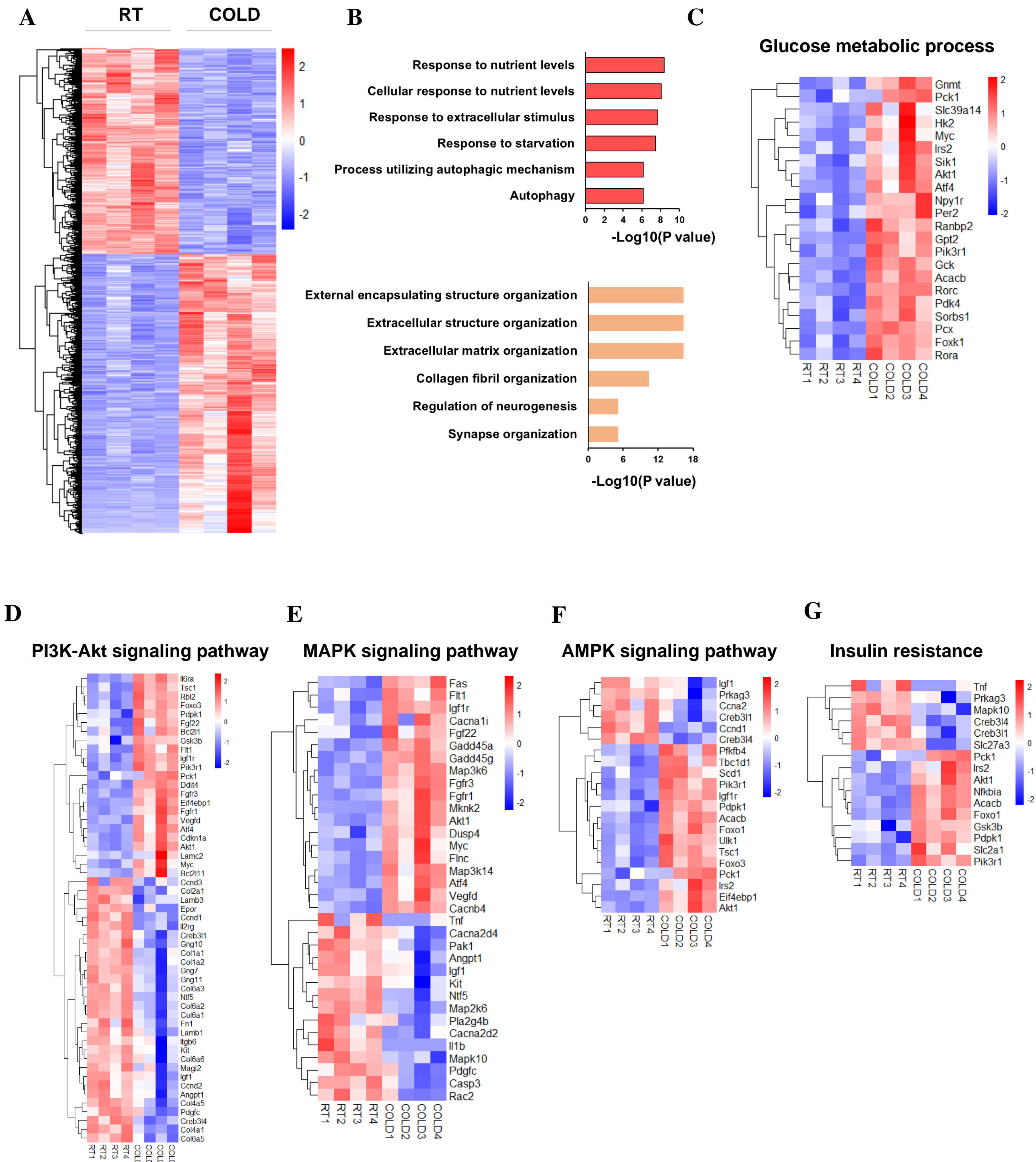

Supplement: Supplementary file 4 — Additional file 4: Fig. S3. Short-term cold exposure induces transcriptome programs alterations. (A) Heatmap showing the differentially expressed genes (padj < 0.05 & Abs (Log2 fold changes) > 1) in TA muscles from control and cold-treated mice (n = 4). Red and blue indicate upregulated differential and downregulated differential expression genes, respectively. (B) Gene Ontology analysis showing the enrichment of functional categories (n = 4). (C) Heatmap showing the differentially expressed genes related to glucose metabolic processes (n = 4). (D-G) Heatmap showing the differentially expressed genes related to PI3K-Akt signaling pathway, MAPK signaling pathway, AMPK signaling pathway and Insulin resistance (n = 4). [file 12915_2023_1514_MOESM4_ESM.pdf]

Fig. S4

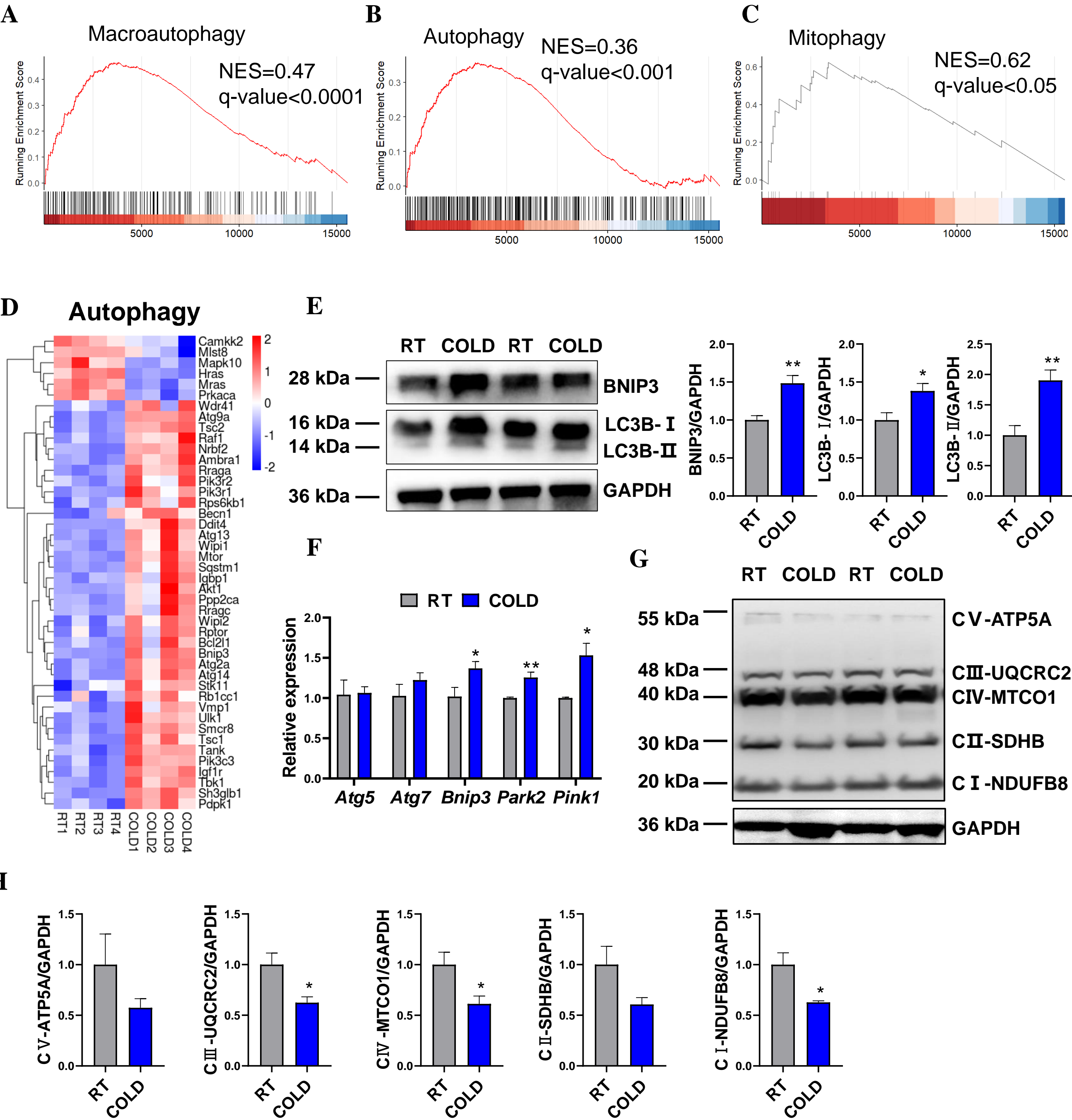

Supplement: Supplementary file 5 — Additional file 5: Fig. S4. Short-term cold exposure induces transcriptome programs alterations. (A-C) Gene set enrichment analysis showing significant enrichment in macroautophagy, autophagy, and mitophagy from control and cold-treated mice. (D) Heatmap showing the differentially expressed genes related to autophagy (padj < 0.05 & Abs (Log2 fold changes) > 1) in TA muscles from control and cold-treated mice. Red and blue indicate upregulated differential and downregulated differential expression genes, respectively (n = 4). (E) Western blot of BNIP3 and LC3B proteins expression in TA muscles from RT and cold-treated mice, GAPDH as loading control. (F) mRNA expression of the autophagy (Atg5 and Atg7) and mitophagy (Pink1, Park2, and Bnip3) genes in TA muscles from RT and cold-treated mice (n = 4). (G-H) Western blot and quantitative analysis of mitochondrial fractions showing oxidative phosphorylation protein in TA muscles from RT and cold-treated mice, GAPDH as loading control. Error bars represent s.e.m.* P < 0.05, ** P < 0.01, *** P < 0.001, two-tailed Student’s t-test. [file 12915_2023_1514_MOESM5_ESM.pdf]
