# Supplementary material for: Cold exposure alters lipid metabolism of skeletal muscle through HIF-1α-induced mitophagy
Source: BMC Biol. 2023 Feb 8;21:27. doi: 10.1186/s12915-023-01514-4 (PMC9906913; doi:10.1186/s12915-023-01514-4)

Fig. S2

A

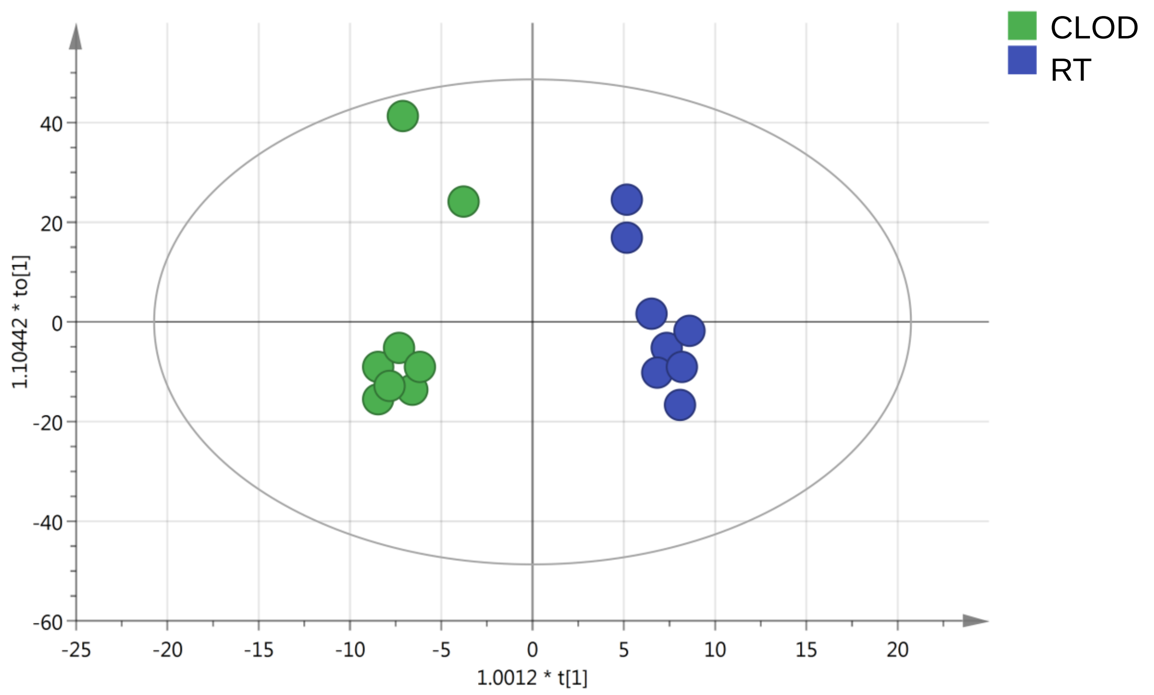

B

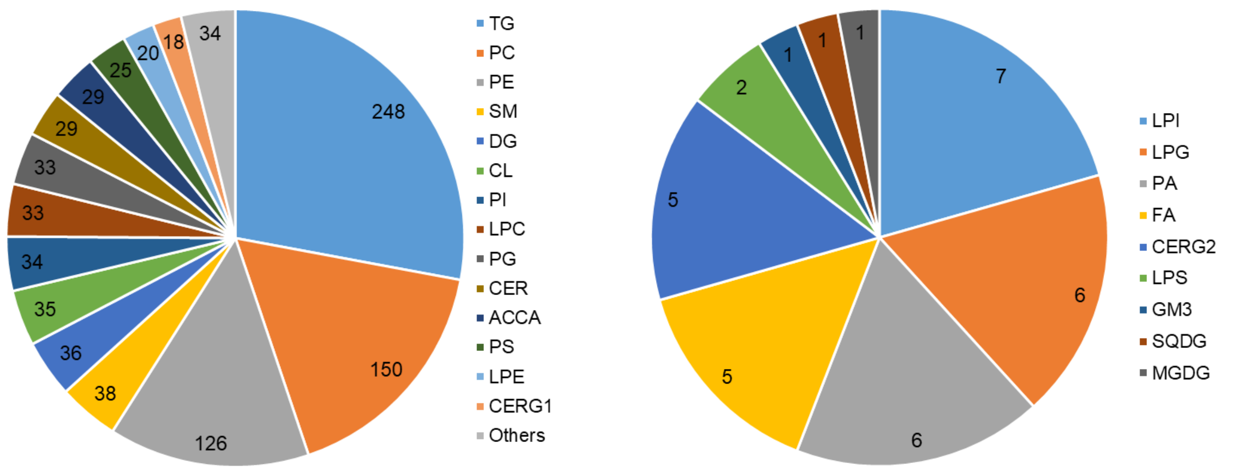

C

Lipid Class	Lipid Name
LPC	Lysophosphatidylcholine
LPE	Lysophosphatidylethanolamine
TG	Triglyceride
PC	Phosphatidylcholine
PE	Phosphatidylethanolamine
SM	Sphingomyelin
DG	Diglyceride
CL	Cardiolipin
PI	Phosphatidylinositol
PG	Phosphatidylglycerol
CER	Ceramides
ACCA	Acyl Carnitine
CERG1	Simple Glc series
PS	Phosphatidylserine
PA	Phosphatidic acid
LPG	Lysophosphatidylglycerol
LPS	Lysophosphatidylserine
FA	Fatty acid
SQDG	Sulfoquinovosyldiacylglycerol
MGDG	Monogalactosyldiacylglycerol
GM3	Gangliosides

D

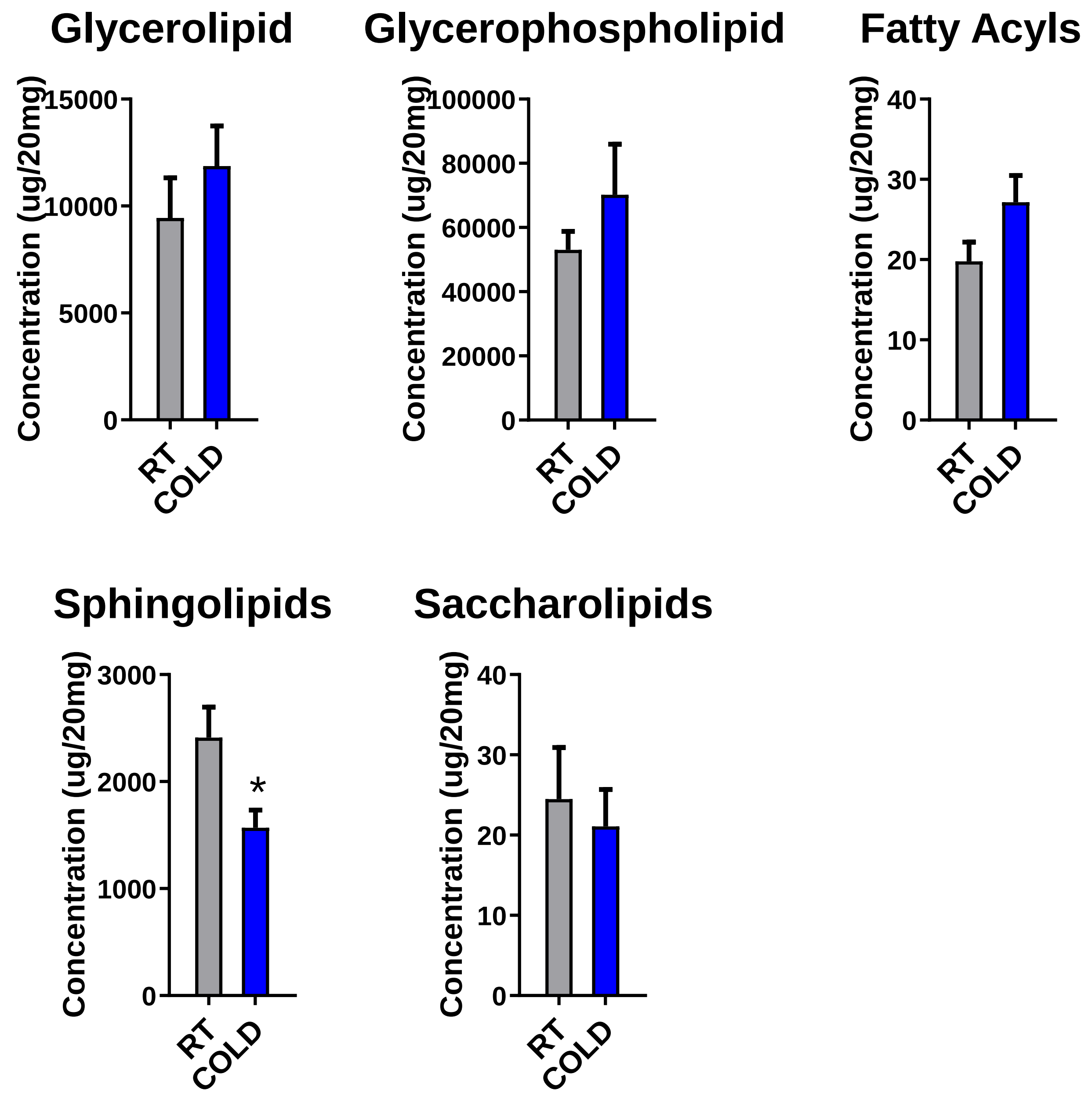

E

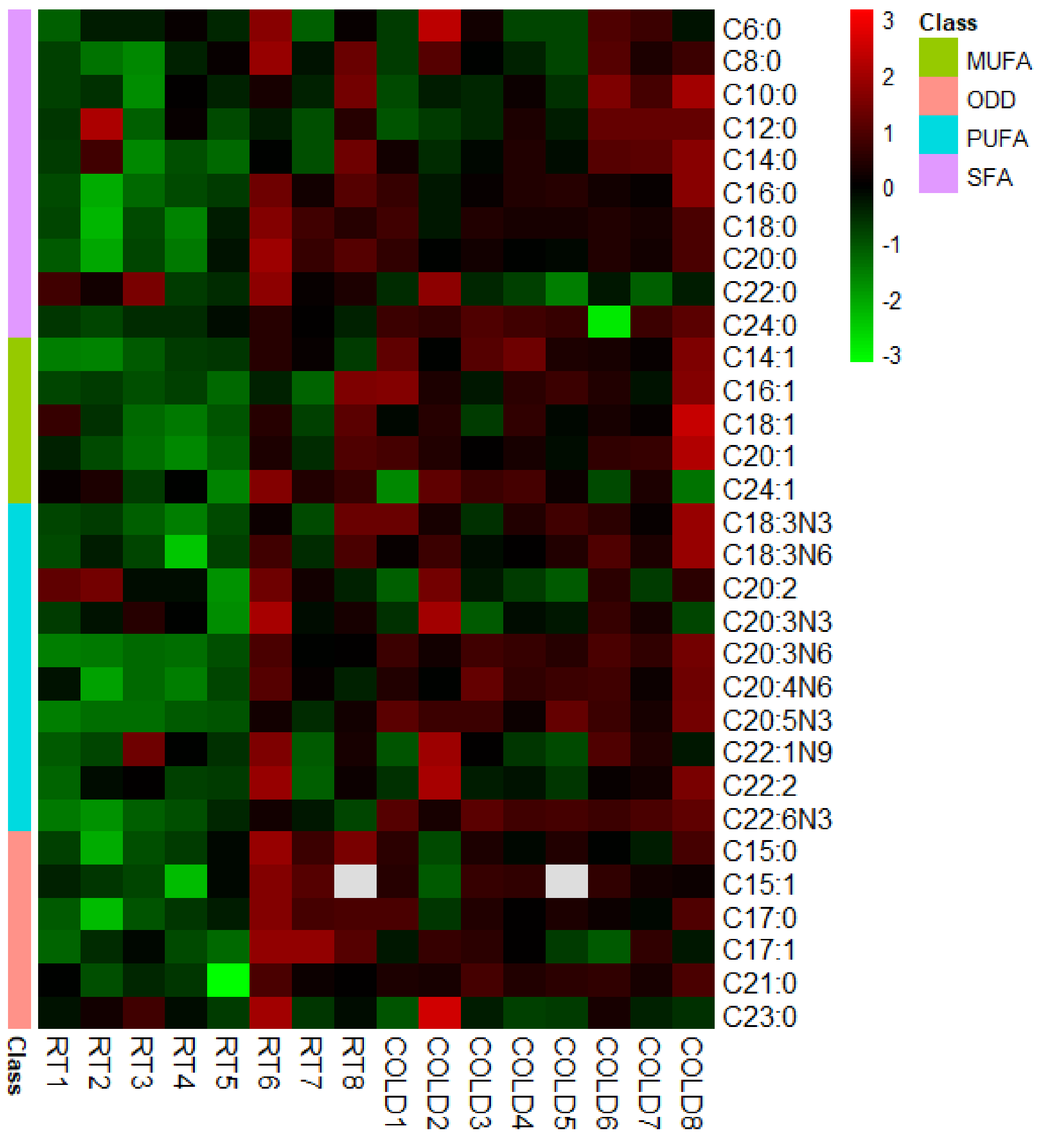

Supplement: Supplementary file 2 — Additional file 2: Fig. S2. Cold exposure alters skeletal muscle lipid composition. (A) OPLS-DA scores plot. Blue and green symbols represent RT and COLD samples, respectively. (B) Composition of lipid classes that were considered for subsequent analysis in all of the samples detected by LC-MS. (C) Quantified lipid classes and their abbreviations. (D) The intensity of glycerolipids, glycerophospholipids, fatty acyls, sphingolipids and saccharolipids in the TA muscle of the RT and COLD group mice. (E) Heatmap showing the total intensity of individual fatty acyl chains altered in COLD vs RT muscle. Error bars represent s.e.m.* P < 0.05, ** P < 0.01, *** P < 0.001, two-tailed Student’s t-test. [file 12915_2023_1514_MOESM2_ESM.pdf]
