# Supplementary material for: Cold exposure alters lipid metabolism of skeletal muscle through HIF-1α-induced mitophagy
Source: BMC Biol. 2023 Feb 8;21:27. doi: 10.1186/s12915-023-01514-4 (PMC9906913; doi:10.1186/s12915-023-01514-4)
Supplement: Supplementary file 8 — Additional file 8. Full scans of immunoblots. [file 12915_2023_1514_MOESM8_ESM.pptx]

## Slide 1
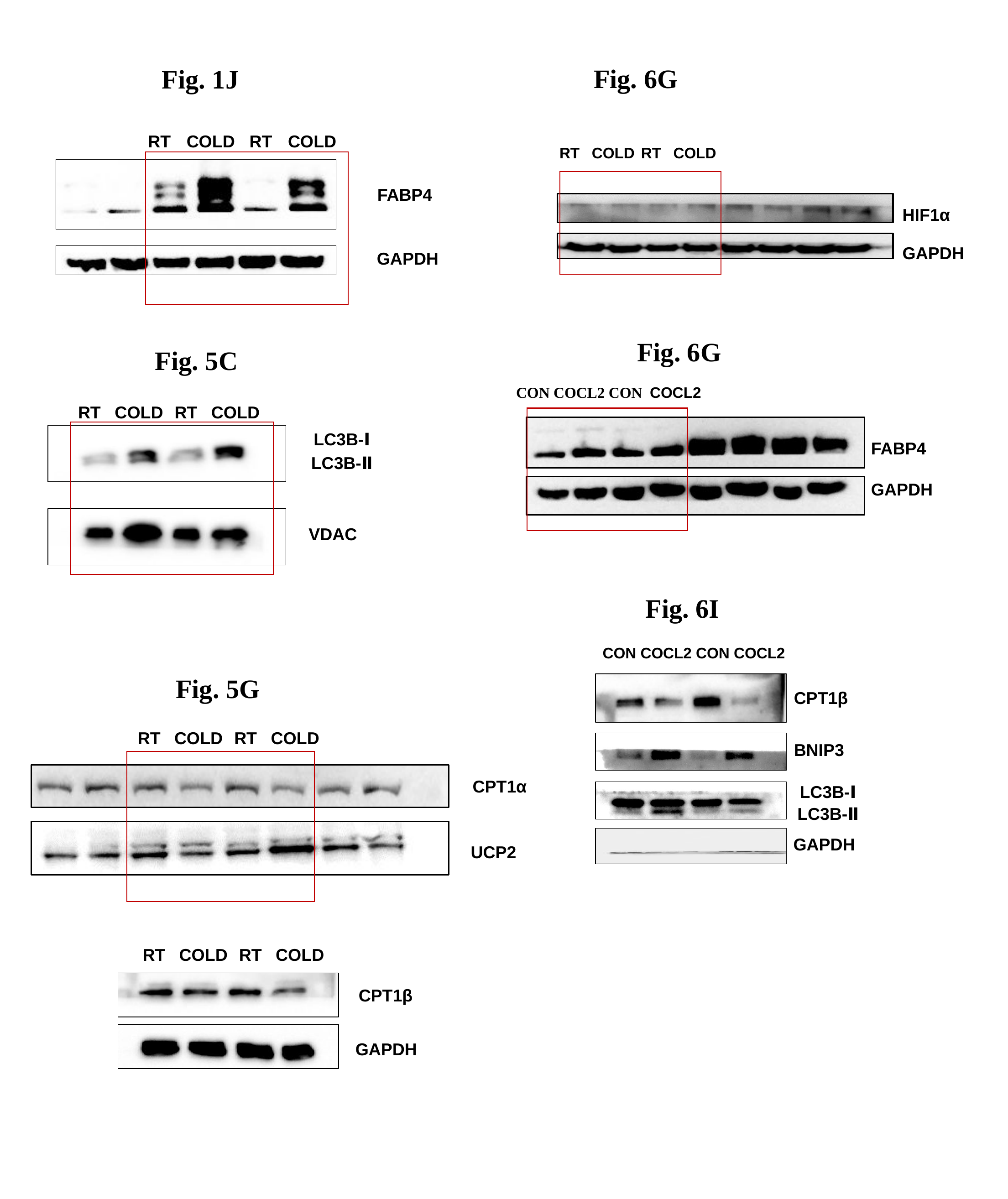

Fig. 6G
Fig. 1J
RT
COLD
RT
COLD
RT
COLD
RT
COLD
FABP4
HIF1α
GAPDH
GAPDH
Fig. 6G
Fig. 5C
CON COCL2 CON COCL2
RT
COLD
RT
COLD
LC3B-Ⅰ
FABP4
LC3B-Ⅱ
GAPDH
VDAC
Fig. 6I
CON COCL2 CON COCL2
Fig. 5G
CPT1β
RT
COLD
RT
COLD
BNIP3
CPT1α
LC3B-Ⅰ
LC3B-Ⅱ
GAPDH
UCP2
RT
COLD
RT
COLD
CPT1β
GAPDH

## Slide 2
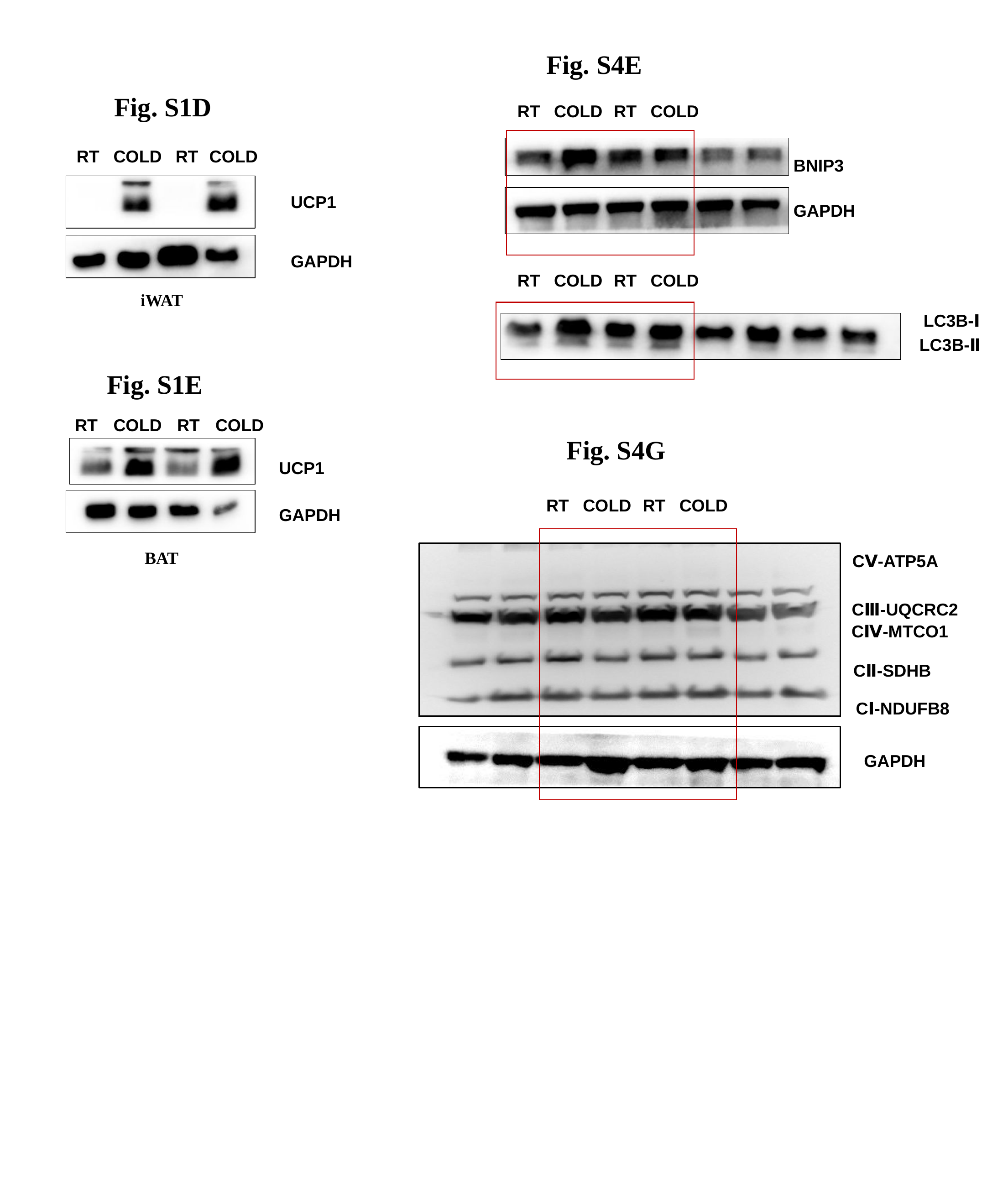

Fig. S4E
Fig. S1D
RT
COLD
RT
COLD
RT
COLD
RT
COLD
BNIP3
UCP1
GAPDH
GAPDH
RT
COLD
RT
COLD
iWAT
LC3B-Ⅰ
LC3B-Ⅱ
Fig. S1E
RT
COLD
RT
COLD
Fig. S4G
UCP1
RT
COLD
RT
COLD
GAPDH
BAT
CⅤ-ATP5A
CⅢ-UQCRC2
CⅣ-MTCO1
CⅡ-SDHB
CⅠ-NDUFB8
GAPDH
